# A new species of *Bestiolina* (Crustacea, Copepoda, Calanoida, Paracalanidae) from coastal waters of the Colombian Pacific, including a worldwide key for the identification of the species

**DOI:** 10.3897/zookeys.846.31497

**Published:** 2019-05-16

**Authors:** John Dorado-Roncancio, Santiago Gaviria, Luis Bernal-De La Torre, Michael J. Ahrens

**Affiliations:** 1 Universidad de Bogota Jorge Tadeo Lozano, Facultad de Ciencias Naturales e Ingeniería, Programa de Ciencias Biológicas y Ambientales, Laboratorio de Limnología, Cra 4 No 22-61, Módulo 5, Piso 8, Bogotá, Colombia Universidad de Bogota Jorge Tadeo Lozano Bogotá Colombia; 2 University of Vienna, Dept of Limnology and Bio-Oceanography and Technisches Büro für Biologie, Fred-Raymond-Gasse 19/2/4, A-1220, Vienna, Austria University of Vienna Vienna Austria; 3 Pontificia Universidad Javeriana, Facultad de estudios ambientales y rurales. Transversal 4° No 42-00, Bogotá, Colombia Pontificia Universidad Javeriana Bogotá Colombia

**Keywords:** Coastal zone, crustaceans, estuaries, taxonomy, tropical zooplankton

## Abstract

Plankton samples obtained from estuarine waters of the Colombian Pacific yielded adults specimens of an undescribed species of a paracalanid copepod of the genus *Bestiolina*. It most closely resembles two Asian species; *B.sinica* (Shen & Lee, 1966) from China and *B.arabica* (Ali, Al-Yamani & Prusova, 2007) from the Arabian Gulf. These three species share the absence of spinules on the posterior surfaces of exopod segments of legs 2, 3 and 4. *Bestiolinasarae* Dorado-Roncancio & Gaviria, **sp. n.** can be easily separated from *B.sinica* by the number of spinules on the anterior surface of endopod 2 of legs 2 and 3, and by the absence of spinules on the posterior surface of second endopod of leg 4. It can be distinguished from *B.arabica* by the presence of spinules on the posterior surface of endopod 2 of same legs (absent in *B.arabica*), and the size of spinules on the anterior surface of the same segments. The only other species known from the Americas, *B.mexicana* (Suárez-Morales & Almeyda-Artigas, 2016), can be distinguished from *Bestiolinasarae* Dorado-Roncancio & Gaviria, **sp. n.** by the presence of spinules on the posterior surface of the leg 2 first exopodal segment and the morphology of the mandible blade. The morphological and meristic differences to the eight known species of the genus are presented. An identification key to the species of *Bestiolina* is provided.

## Introduction

The family Paracalanidae is represented by seven genera (Razouls et al. 2018; Walter and Boxshall 2018) and are among the common estuarine and coastal planktonic copepods of tropical and subtropical latitudes (Suárez-Morales and Almeyda-Artigas 2016). The paracalanid genus *Bestiolina* (Andronov 1991) currently includes eight species and was originally named *Bestiola* (Andronov 1972). It was subsequently renamed because *Bestiola* was preoccupied by an insect generic name (Nikolskaya 1963). It can be considered as a relatively recently described genus in relation to the first descriptions of marine planktonic copepod species done at the middle of the 19^th^ century (i.e., Dana 1852; Claus 1863; Boeck 1865; Brady 1899). Most species of *Bestiolina* were described in the last 20 years (Mulyadi 2004; Ali et al. 2007; Moon et al. 2010; Suárez-Morales and Almeyda-Artigas 2016). The poor knowledge of the diversity and distributional patterns of the genus could be explained by their small size (670–1008 µm), inappropriate sampling techniques (nets with mesh size > 200 µm) and confusion with copepodite stages of other paracalanid species. The lack of information about *Bestiolina* in the tropical eastern Pacific could also be explained by the few faunal surveys done in coastal waters of the region.

*Bestiolina* can be characterized as a coastal-neritic copepod genus that lives in shallow waters near the coastal areas (Bradford-Grieve 1994; Boxshall and Hasley 2004). Species of *Bestiolina* are concentrated in tropical latitudes of different oceans, and its origin has been speculated to be Indo-Malayan (Ali et al. 2007). Except for the record of *Bestiolinamexicana* in the Gulf of Mexico (Suárez-Morales and Almeyda-Artigas 2016), no other species of *Bestiolina* have been hitherto recorded in coastal waters of the Americas.

During the development of a project to evaluate marine bioinvasions in the Colombian Pacific and their relation with marine traffic, zooplankton samples were collected in three different coastal areas. Specimens of *Bestiolina* present in several samples could not be assigned to any known species of the genus and was thus deemed as new. Based on several adult female and male specimens available, the species is described and illustrated herein.

## Methods

**Figure 1.**
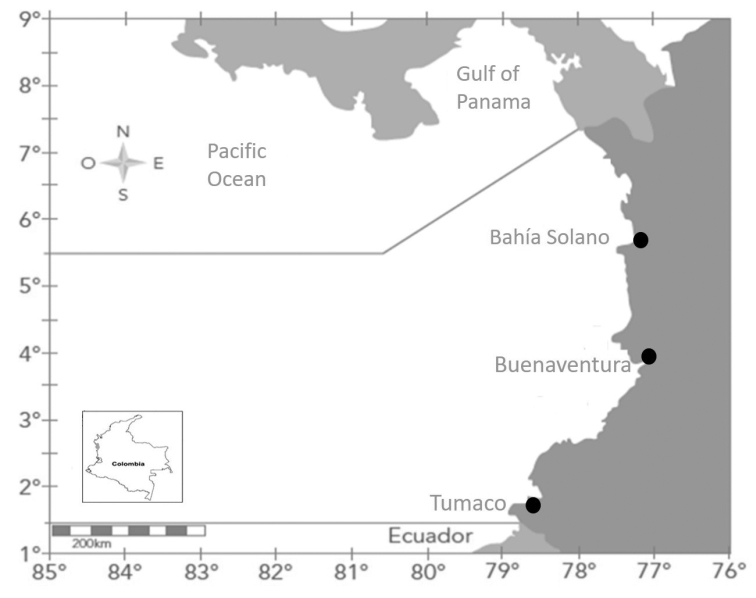
Sampling locations (modified from Dorado Roncancio 2018).

Zooplankton samples were collected only once in six localities from three major port areas of the Colombian Pacific coast (Fig. 1) between September/October 2016 and May/June 2017 as follows:

1) Bahia Solano, Departamento del Chocó (6°14'N, 77°24'W)

2) Huina, Departamento del Chocó (6°16'N, 77°27'W)

3) Buenaventura, Departamento del Valle del Cauca (3°53'N, 77°03'W)

4) Bahia de Málaga-Juanchaco, Departamento del Valle del Cauca (3°55'N, 77°20'W)

5) Tumaco Port, Departamento de Nariño (1°48'N, 78°45'W)

6) Tumaco City, Departamento de Nariño (1°49'N, 78°45'W)

Samples were obtained via surface trawls using a standard zooplankton net with 150 µm mesh size hauled for 2 minutes from a boat travelling at approximately 2 knots. Zooplankton was narcotized with MgCl_2_ (Suther and Rissik 2009) prior to fixation and preservation in ethanol 80% at a 1:3 ratio.

Dissection techniques followed Björnberg (1981). Specimens were dissected in glycerine using sharpened tungsten needles. Specimens were then mounted in Entellan (J. Dorado) and lactophenol (S. Gaviria) and sealed with varnish. Animals were studied using a Zeiss Ax10 Scope A1 (J. Dorado) and a Nikon Ellypse 200 (S. Gaviria). Drawings were performed based on images obtained with a Zeiss Ax10 Scope equipped with a digital camera.

Type specimens were deposited at the Museo de Historia Natural Marina de Colombia, Santa Marta, Colombia (MAKURIWA) of the Instituto de Investigaciones Marinas y Costeras INVEMAR, and at the Naturhistorisches Museum Wien (NHMW) in Vienna, Austria.

The descriptive terminology follows Huys and Boxshall (1991) and Ferrari and Ivanenko (2008).

Environmental parameters were measured *in situ* with a multiparametric probe (Hach-HQ40d) and water transparency was determined with a Secchi disk. At each site, 200 ml water was filtered through glass fibre filters (Whatman GFC) for chlorophyll-*a* analysis (*ex situ* using spectrophotometry). Water temperature, salinity, dissolved oxygen, Secchi depth and chlorophyll-*a* data, together with standard deviation (SD) were as follows: surface water temperature x̄ = 28.7 °C (SD 1.0 °C, *n* = 18) in 2016 and x̄ = 28.8 °C (SD 1.1 °C, *n* = 14) in 2017; salinity x̄ = 23.0 (SD 6.1, *n* = 18) in 2016 and x̄ = 23.9 (SD 1.1, *n* = 14) in 2017; dissolved oxygen x̄ = 6.4 mg/L (SD 0.6 mg/L, *n* = 18) in 2016 and x̄ = 6.7 mg/L (SD 0.5 mg/L, *n* = 14) in 2017. Secchi depth was x̄ = 3.6 m (SD 3.5 m, *n* = 18) in 2016 and x̄ = 4.8 m (SD 3.9 m, *n* = 14) in 2017. Chlorophyll-*a* concentration was x̄ = 3.0 µg/L (SD 4.3 µg/L, *n* = 17) in 2016 and x̄= 2.8 µg/L (SD 4.1 µg/L, *n* = 19) in 2017.

## Results

### Taxonomy

#### Class Hexanauplia Oakley, Wolfe, Lindgren & Zaharof, 2013

##### Subclass Copepoda Milne Edwards, 1840

###### Order Calanoida G.O. Sars, 1903

####### Family Paracalanidae Giesbrecht, 1893

######## Genus *Bestiolina* Andronov, 1991

######### Bestiolina sarae

Taxon classificationAnimaliaCalanoidaParacalanidae

Dorado-Roncancio & Gaviria sp. n.

http://zoobank.org/E0A2340A-31B3-42B2-BB70-418C4DFBA9A4

########## Material examined.

Holotype: Adult female (MAKURIWA INV-CRU8991) dissected on a slide, mounted in Entellan. Allotype: male dissected on a slide (MAKURIWA INV-CRU8992), mounted in Entellan. Paratypes: two females (NHMW 26309 and 26310), each one dissected on three slides and mounted in lactophenol, one female (NHMW 26311) dissected and mounted in one slide; six females (NHMW 26312) undissected and preserved in ethanol; one female and two males undissected, preserved in ethanol+glycerine (MAKURIWA INV-CRU8993 y 8994). Material was collected by L. Bernal, M. Ahrens and J. Dorado-Roncancio, as follows: holotype and allotype on 30/09/2016 near Buenaventura harbor (03°53'49.054"N, 077°03'44.3"W), paratypes on 26/07/2017 in the Bahía Málaga (03°55'30.759"N, 077°20'56.48"W).

########## Etymology.

The new species is named in honour of Sara Dorado, an important member of the family of the first author, who passed away one year before the discovery of the species. The name of the species is a feminine noun in genitive singular.

########## Type locality.

Near Buenaventura harbor (03°53'49.054"N; 077°03'44.3"W) (Fig. 1), Eastern Pacific Ocean, Colombia. At the type locality, the waters are characterized as coastal and estuarine. The type locality belongs to the Buenaventura natural ecoregion of the Colombian Pacific according to the classification of Diaz and Acero (2003). The area is characterized by bays, with an average depth between 12 m and 15 m, and tectonic estuaries, which include a wide variety of habitats such as sandy and rocky beaches, mudflats, large areas of high-productivity mangroves, sandstone cliffs and soft-sediment floodplains. Many rivers and streams empty into the sea, bringing high amounts of sediments and causing variations in the physical and chemical conditions of the waters (Lazarus-Agudelo et al. 2007, Betancourt and Portela et al. 2011). Precipitation in the region is very high (> 5000 mm/y) (Dimar 2002). Water chemistry can be characterized as follows: surface temperature ranges between 26.6 °C and 29.7 °C; salinity between 1.3 and 30 psu; relative humidity close to 90%. Precipitation for the area ranges between 5000–7000 mm per year, semidiurnal tides with an average range of 4.1 m (Cantera and Blanco 2001).

########## Differential diagnosis.

*Bestiolina* of small size (female 0.64–0.73 mm, male 0.63–0.75 mm), with body divided in prosome and slender urosome. Cephalic dorsal hump present in male. Rostrum short and stout divided in acute points. First pedigerous somite fused with cephalothorax, fifth pedigerous somite separated from preceding somite. Posterolateral margins of fifth pedigerous somite rounded and ornamented with small spinules. Genital double-somite with ventral protuberance in adult females. Exopods of legs 2–4 with anterior and posterior surfaces of all segments without spinules. Endopod 2 of legs 2 and 3 with anterior surface mostly with 3 small spinules and posterior surface mostly with 4 large spinules. Leg 5 of female rudimentary, unsegmented, consisting of a pair of rounded lobes, lobes with smooth margin. Leg 5 of male asymmetrical, right leg as in female, left leg long, 5-segmented, last segment with long distal spine.

########## Description of holotype female.

**Figure 2.**
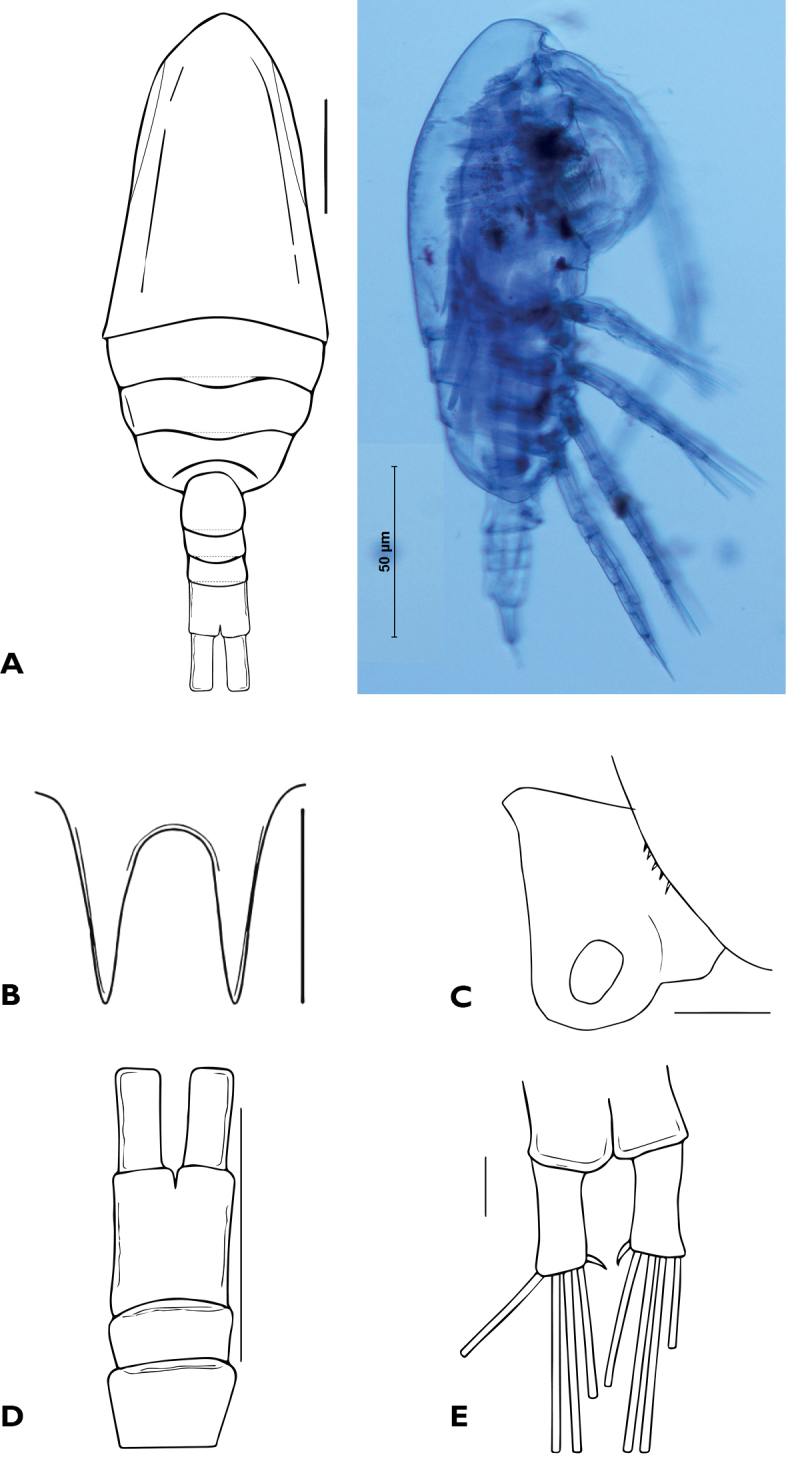
Female Holotype of *Bestiolinasarae* sp. n. **A** habitus, dorsal view and digital photograph **B** rostrum **C** posterolateral margins of fifth pedigerous somite, lateral view **D** second and third urosomites and anal somite with caudal rami **E** caudal rami and setae. Scales bar: 0.1 mm (**A**, **D**); 0.01 mm (**B**, **E**); 0.05 mm (**C**).

(Fig. 2A) Length of specimen measured from tip of rostrum to posterior margin of caudal rami: 0.70 mm. Body robust, widest section at second somite, anterior part of cephalosome rounded. Rostrum short and stout, divided into acute points (Fig. 2B). First pedigerous somite completely fused with cephalosome. Second, thirth and fourth pedigerous somites free. Fifth pedigerous somite completely separated from fourth, with posterolateral margins rounded and bearing small spinules (Fig. 2C).

Urosome, 4-segmented. First and second urosomites fused forming a ventrally expanded genital-double somite. Anal somite slightly longer than second and third urosomites together (Fig. 2D). Caudal rami not divergent, shorter than anal somite, armed with 5 setae. Dorsal setae (VII) strongly reduced, setae I and II lacking (Huys and Boxshall 1991). Without setae on the inner and outer sides of rami (Fig. 2E).

**Figure 3.**
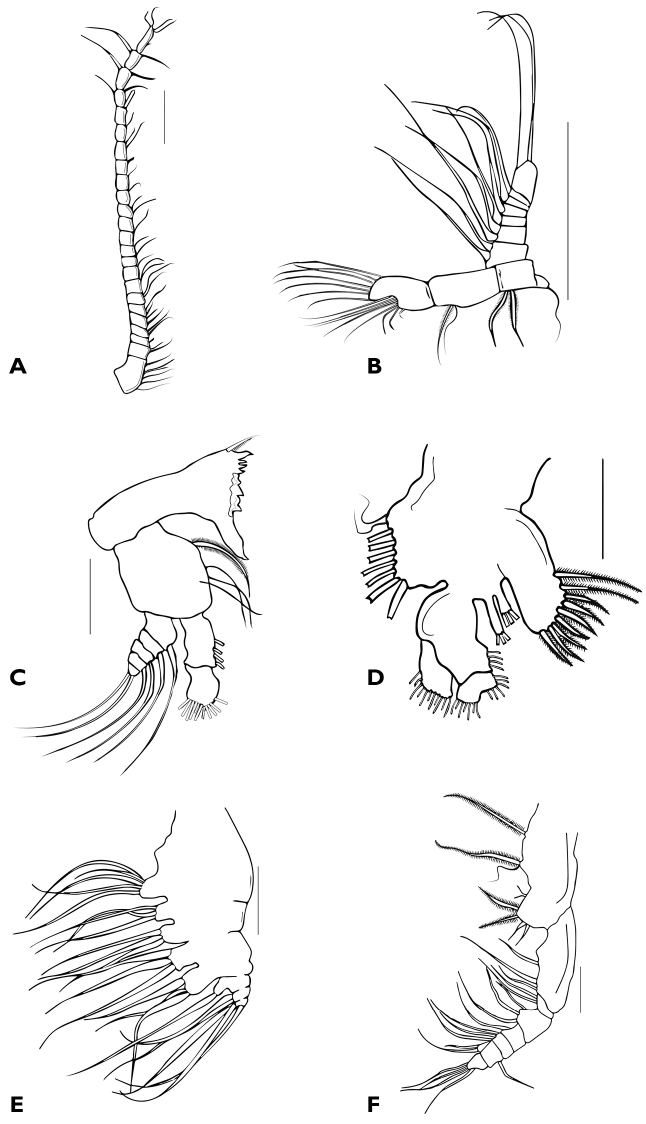
Female Holotype of *Bestiolinasarae* sp. n. **A** antennule **B** antenna **C** mandible **D** maxillule **E** maxilla **F** maxilliped. Scales bars: 0.1 mm (**A**); 0.05 mm (**B–F**).

Antennule 24-segmented (Fig. 3A). Ancestral segments (Huys and Boxshall 1991) I – IV and XXVII–XXVIII fused. Armature formula with current segments designated with Arabic numerals (s = seta, sp = spine, ae = aesthetask): 1:6s, 2:2s, 3:1s, 4:2s, 5:1s, 6–8:2s, 9–12:1s, 13:0s, 14:2s, 15:1s, 16:1s, 17:1ae, 18:2s, 19:1s, 20:1ae, 21 to 23:2s, 24:4s+1sp.

Antenna (Fig. 3B) biramous. Coxa small, partially fused with basis, with 1 seta. Basis with 2 long distal seate. Endopod 2-segmented, first segment with 2 subdistal setae, second segment bilobated, subterminal lobe with 8 setae, terminal lobe with 7 setae. Exopod 7-segmented, first and second segments fused, each with 2 setae, segments 3–6 each with 1 seta, terminal segment with 3 setae.

Mandible (Fig. 3C) with thick gnathobase armed with 3 medial teeth, 4 dorsal teeth, 1 large anterior tooth separated from main cuting edge by a diastemma, and a short dorsal seta. Palp basis with 4 subequal setae; endopod 2-segmented, first segment with 4 distal setae, second segment with 11 subequal setae; exopod short, 5-segmented, each segment with 1 seta except distal segment with 2.

The maxillule, maxilla and maxilliped are described according to Ferrari and Ivanenko (2008).

Maxillule (Fig. 3D) with precoxal endite bearing 9 thick spiniform setae. Coxa with 2 endites, each endite with 3 setae, exite with 9 setae. Basis with 4 setae on inner margin; distal endite bilobated with 6 setae on short lobe and 7 setae on long lobe. Exopod with 11 setae.

Maxilla (Fig. 3E), precoxal endite of syncoxa armed with 5 setae. Three coxal endites each with 3 setae. Basis with 3 setae. Endopod 3-segmented, first segment with endite bearing 1 seta, second segment with 2 setae, third segment with 3 setae.

Maxilliped (Fig. 3F) long. Coxa armed with 4 groups of elements: proximal endite of praecoxa reduced to 1 thick seta, middle endite of praecoxa represented by 1 thick and 1 thin seta, distal endite of praecoxa consists of 2 thin setae, endite of coxa represented by 4 subequal setae. Distal endite of basis with 3 setae. Endopod 6-segmented, setal formula of first 4 segments 2, 3, 1, 3, fifth segment bilobated with 1 and 2 setae on each lobe (each side), distal segment with 4 setae.

**Figure 4.**
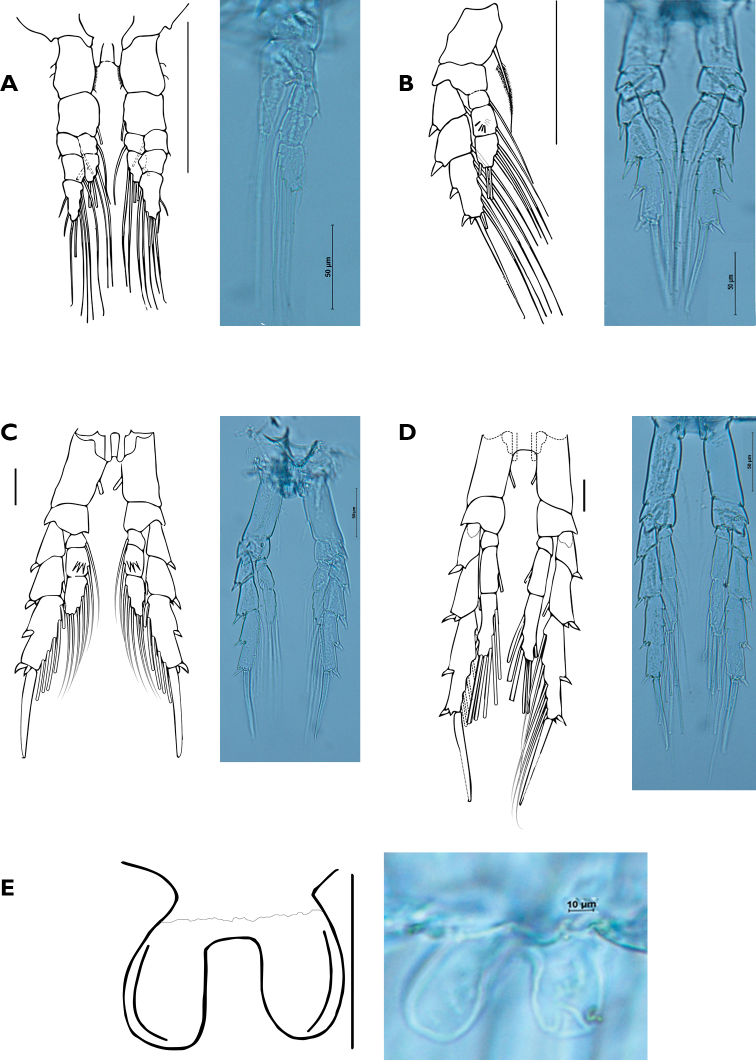
Female of *Bestiolinasarae* sp. n. **A** Leg 1, anterior view, Leg 1 and digital photograph **B** leg 2, posterior view and digital photograph **C** leg 3, anterior view (spinules on posterior surface not indicated in contrast with leg 2) and digital photograph **D** leg 4, anterior view and digital photograph **E** leg 5 and digital photograph. Scales bars: 0.05 mm (**A–D**); 0.01 mm (**E**).

Leg 1 (Fig. 4A): coxa with row of short setae on inner margin, and 2 setae on outer margin. Basis with 1 seta on inner margin. Exopod 3-segmented; first segment with 1 spine distally on outer margin, inner margin with 1 seta; second segment, outer margin naked, inner margin with 1 seta; third segment outer margin with 2 setae, distal margin with 1 seta, inner margin with 4 setae. Endopod 2-segmented; first segment, inner margin with 1 seta; second segment, outer margin with 1 seta, distal margin and inner margins each with 2 setae. Anterior and posterior surfaces of all segments without spinules.

Legs 2 to 4 (Fig. 4B–D): coxa with 1 seta on inner margin. Basis without marginal seta. Legs with 3-segmented exopod and endopod. Exopod, first and second segments, outer margin with 1 short and thick distal spine, first segment inner margin with 1 seta (leg 2) or without seta (leg 3 and 4); third segment, outer margin with 3 short and thick spines, 1 inserted medially, 2 inserted subapically, distal margin with 1 long spine, inner margin with 5 setae. Long spine of distal margin thicker on leg 3 and 4.

Leg 2 (Fig. 4B): exopod, anterior and posterior surfaces of all 3 segments without spinules. Endopod, anterior and posterior surfaces of first and third segment without spinules, second segment, anterior surface with 3 short spinules, posterior surface with 4 long spinules.

Leg 3 (Fig. 4C): number and size of spinules of anterior and posterior surfaces like leg 2. Distal segment of endopods of legs 3 and 4 with 6 setae.

Leg 4 (Fig. 4D): exopod and endopod, anterior and posterior surfaces of all segments without spinules.

Leg 5 (Fig. 4E): reduced in size, represented by symmetrical lobes with smooth margins.

Spine (Roman numerals) and setal (Arabic numerals) formula of legs 1–4 as follows:

**Table:** 

**Leg**	**Coxa**	**Basis**	**Exopod**	**Endopod**
**1**	0–0	0–1	0–1; 0–1; 2,1,4	0–1; 1,2,2
**2**	0–1	0–0	I–1; I–1; III,I,5	0–1; 0–1; 1,2,3
**3**	0–1	0–0	I–0; I–1; III,I,5	0–1; 0–1; 1,2,3
**4**	0–1	0–0	I–0; I–1; III,I,5	0–0; 0–1; 1,2,3

**Figure 5.**
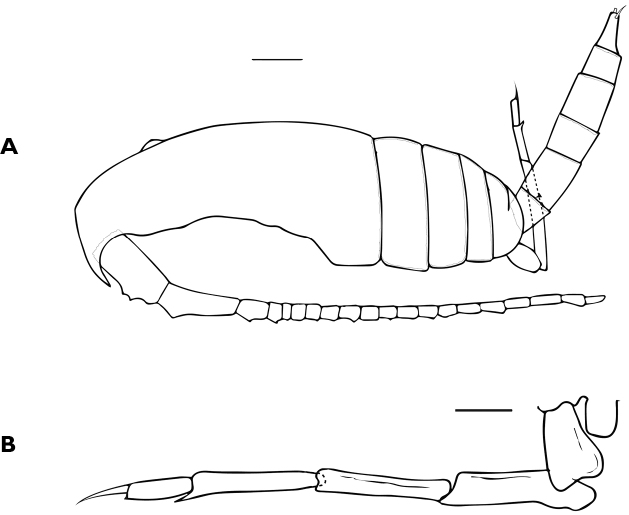
Male of *Bestiolinasarae* sp. n. **A** Habitus, lateral view **B** leg 5. Scales bars: 0.1 mm (**A**); 0.01 mm (**B**).

Description of male (Fig. 5A): length of allotype measured from tip of rostrum to tip of caudal rami: 0.70 mm. Body more slender and slightly longer than in female. Cephalothorax with dorsal hump. Antennule 20-segmented, setation patterns of ancestral segments (indicated with Arabic numerals, s = seta), as follow: 1:3s, 2:1s, 3:1s, 4:1s, 5 and 8:0s, 9:1s, 10 and 12:0s, 13:1s, 14 and 15:0s, 16:1s, 17:0s, 18:1s, 19:1s, 20:4s.

First to fifth pedigerous somites and swimming legs like in female. Urosome 5-segmented. Leg 5 (Fig. 5B) typical for the family, right leg consisting of a rounded lobe as in female, left leg elongate, 5-segmented, distal segment with apical spine.

Variability (Table 1): Females (*n* = 13): morphological variability in body length x̄ = 0.70 ± 0.03 (0.64–0.73 mm) and in ornamentation pattern (number of spinules) of endopod 2 of legs 2 and 3.

Leg 2, endopod 2: holotype and 1 paratype (NHMW 26311) with 3 short spinules on anterior surface and 4 long spinules on posterior surface of left and right legs, one paratype (NHMW 26310) shows an additional spinule on anterior surface (4 instead of 3) of right leg, one paratype (NHMW 26311) shows an additional spinule on posterior surface of same leg. Two paratypes (MAKURIWA INV-CRU8993, NHMW 26309) show a different combination of spinules: one additional spinule on the anterior surface of both legs (left and right) (4 instead 3) and 1 additional spinule on posterior surface of left and right legs (5 instead of 4) MAKURIWA (Table 1).

Leg 3, endopod 2: variability of the ornamentation pattern was also noted on this leg but less accentuated than in leg 2 (Table 1). Anterior surface with 3 short spinules on anterior surface and 4 long spinules on posterior surface (holotype and paratype NHMW 26310) on both left and right legs; one paratype (MAKURIWA INV-CRU8993) shows 1 additional spinule on both legs on anterior and posterior surfaces (4+5 instead of 3+4). One paratype (NHMW 26309) shows 1 additional spinule on posterior surface (total 3+5) of left leg. In general, the most common spinulation pattern of second endopods in legs 2 and 3 is 3 spinules on anterior surface and 4 on posterior surface.

Males (*n* = 3) show variability on body length x̄ = 0.70 ± 0.06 (0.63–0.75 mm). No variability was noted on spinulation pattern of the 3 studied specimens.

**Table 1.** Number of spinules on anterior and posterior surface of endopod 2 of leg 2 and leg 3 of females of *Bestiolinasarae* sp. n. (holotype and four paratypes). Spinules of anterior surface are small, spinules of posterior surface are large and strong. n/o means spinules not observed (segment lost during dissection).

Character		Holotype MAKURIWA INV-CRU8991	Paratype MAKURIWA INV-CRU8993	Paratype NHMW 26309	Paratype NHMW 26310	Paratype NHMW 26311
Number of spinules anterior + posterior surface	Leg 2 left	3+4	4+5	4+4	3+4	3+4
	Leg 2 right	3+4	4+5	4+4	4+4	3+5
	Leg 3 left	3+4	4+5	3+5	3+4	n/o+n/o
	Leg 3 right	3+4	4+5	3+4	3+4	n/o+n/o

## Discussion

Specimens from the Colombian Pacific were identified as belonging to the genus *Bestiolina* based on the diagnostic characters of the genus (Bradford-Grieve 1994): relatively short rostrum, presence of one seta on the inner margin of basis of leg 1, outer margin of exopodal segments 2 and 3 of legs 2–4 without teeth, and distal segment of endopods of legs 3 and 4 with 6 setae. The typically reduced female fifth leg, with a bilobated form in female, the dorsal hump of cephalothorax and the asymmetrical legs 5 with long left leg and bilobated right leg in male, constitute the most discriminative characteristics of *Bestiolina*.

Adult members of genus *Bestiolina* can be confused with juvenile stages of other Paracalanidae due to the size of the anal segment (slightly longer than urosomites 2 and 3 together). Copepodites V of the other Paracalanidae have the same pattern and only the adult stages show an anal segment as long as urosomite 3. Additionally, the morphology of female leg 5 in immature stages of *Acrocalanus* and *Parvocalanus* is similar to adult stages of females of *Bestiolina*.

Specimens of Colombian *Bestiolina* were compared with the eight known species of the genus (Tables 2, 3).

*Bestiolinasarae* sp. n. can be distinguished from the other species by a combination of morphological characters related to body length, the number of segments of the antennule, the relationship of first pedigerous somite to cephalosome, and ornamentation of fifth pedigerous somite (Table 2). The three species, *Bestiolinazeylonica* (Andronov, 1972), *Bestiolinamexicana* (Suárez-Morales & Almeyda-Artigas, 2016) and *Bestiolinasarae* sp. n., are the smallest of the genus. The number of segments of the antennule is 25 in *Bestiolinacoreana* (Moon, Lee & Soh, 2010) and *Bestiolinasimilis* (Sewell, 1914), 24 in *Bestiolinaamoyensis* (Li & Huang, 1984) and *Bestiolinasarae* sp. n., and 23 in the remaining species; no information of this character is available for *Bestiolinasinica* (Shen & Lee, 1966). The cephalosome and the first pedigerous somite are fused in *B.coreana*, *B.similis*, *B.amoyensis*, *Bestiolinaarabica* (Ali, Al-Yamani & Prusova, 2007), *B.sinica*, *B.zeylonica* (Andronov, 1972) and *Bestiolinasarae* sp. n. (Fig. 2A), while they are separate in *B.inermis* (Sewell, 1912) and *B.mexicana*. Three species *B.similis*, *B.arabica* and *B.inermis* have no spinules on the distal margin of the fifth pedigerous somite, while spinules are present in the other species and in *Bestiolinasarae* sp. n. (Fig. 2D).

**Table 2.** Distribution and comparison of female morphological traits related to habitus, antennules, cephalosome and prosome of *Bestiolina* species. n/a no information available.

	*B.coreana* (Moon et al., 2016)	*B.similis* (Sewell, 1914)	*B.amoyensis* (Li & Huang, 1984)	*B.arabica* (Ali et al., 2007)	*B.inermis* (Sewell, 1912)	*B.sinica* (Shen & Lee, 1966)	*B.zeylonica* (Andronov, 1972)	*B.mexicana* (Suárez-Morales & Almeida-Artigas, 2016)	*B.sarae* sp. n.
Distribution	Yellow Sea and Southern waters of Korea	Pacific and Indian Oceans, tropical / subtropical	South China Sea	Arabian Gulf	Pacific and Indian Oceans, tropical / subtropical	South China Sea	Sri Lanka	Gulf of Mexico	Pacific Coast of Colombia
Body, mean length (mm)	0.94	1.08	0.93	0.85	1.08	0.99	0.69	0.67	0.70
Antennule, # of segments	25	25	24	23	23	n/a	23	23	24
Cephalosome and first pedigerous segment	fused	fused	fused	fused	separate	fused	fused	separate	fused
Fifth pedigerous somite, distal margin with spinules	yes	no	yes	no	no	yes	yes	yes	yes

In *Bestiolina*, the ornamentation of endopods and exopods of legs 2–4 is important to distinguish the species (Table 3). *Bestiolinasarae* sp. n. shares with *B.sinica* and *B.arabica* the absence of spinules on anterior and posterior surfaces on exopod segments of legs 2–4, but females of *B.sinica* are longer (x̄ = 0.94 mm) than *Bestiolinasarae* sp. n. (x̄ = 0.70 mm, Table 2), and have 4 and 5 spinules on the anterior surface of endopod 2 of legs 2 and 3, respectively (vs 3 and 3, respectively, in *Bestiolinasarae* sp. n.; Table 3). The new species also shares with *B.arabica* the number of spinules (3) on the anterior surface of endopod 2 of legs 2 and 3, but this species lacks spinules on the posterior surface of the same segments (vs 4 in *Bestiolinasarae* sp. n.). Additionally, spinules of the anterior surface of this segment in *B.arabica* are large while those of *Bestiolinasarae* sp. n. of the same surface are small.

**Table 3.** Comparison of female morphological traits of *Bestiolina* species relatively to ornamentation of exopod (segments 1–3) and endopod (segment 2) of leg 2–4. n/a no information available.

Character	Leg	* B. coreana *	* B. similis *	* B. amoyensis *	* B. arabica *	* B. inermis *	* B. sinica *	* B. zeylonica *	* B. mexicana *	*B.sarae* sp. n.
Exopod, number of spinules on posterior surface of segments 1–3	leg 2 leg 3 leg 4	0,6,3 0,4,0 0,3,0	0,0,3 0,0,3 absent	2,1,1 1,1,2 1,2,1	absent absent absent	0,3,0 n/a n/a	absent absent absent	3,3,2 0,3,2 absent	3,0,0 absent absent	absent absent absent
Endopod 2, number of spinules on anterior and posterior surface of segments 1–3	leg 2 leg 3 leg 4	4+3 4+3 0+4	0+5 0+5 absent	0+5 0+4 0+small spinules	3+0 3+0 absent	4 n/a n/a	4+4 5+4 0+4	4 4+3 0+small spinules	2+4 2+0 3+0	3+4 3+4 absent

The most relevant character to distinguish species of *Bestiolina* is the spinulation pattern on the anterior and posterior surfaces of endopod 2 of legs 2 and 3. *B.sarae* sp. n. bears 3 (anterior surface) and 4 (posterior surface) spinules, where as all other species have a different combination pattern: 4+3 in *B.coreana*, 0+5 in *B.similis*, 0+4 (leg 2) and 0+5 (leg 3) in *B.amoyensis*, 3+0 in *B.arabica*, 4+4 (leg 2) and 4+5 (leg 3) in *B.sinica*, 4+0 (leg 2) and 4+3 (leg 3) in *B.zeylonica*, 2+4 (leg 2) and 2+0 (leg 3) in *B.mexicana*. Although no information is available for leg 3 of *B.inermis* and that it is not specified if the four spinules of leg 2 are inserted at the anterior or posterior surface, other characters like the presence of 3 spinules on the posterior surface of exopod 2 distinguish it from *Bestiolinasarae* sp. n. (no spinules on exopodal segments).

Differences with *B.mexicana* are the form of the rostrum, which is short and stout in *Bestiolinasarae* sp. n. and long and with slender filaments in *B.mexicana*, and the morphology of the cutting edge of the mandible (two dorsal teeth in *B.mexicana*, one in *Bestiolinasarae* sp. n.). Additionally, *B.mexicana* bears spinules on the posterior surface of first exopod segment of leg 2, while this surface is naked in *Bestiolinasarae* sp. n. (Table 2).

Although a high variability on the spinulation pattern of the endopod 2 of legs 2 and 3 was observed in *Bestiolinasarae* sp. n. (Table 1), sometimes also differing between right and left legs of the same individual, the most common pattern is represented by 3+4 (three small spinules on the anterior surface and four large spinules on the posterior surface). Other spinulation patterns observed on the same segments are: leg 2 (4+4, 4+5) and leg 3 (3+5, 4+5). In contrast, *B.arabica* with leg 2 (3+0) and leg 3 (3+0) does not show this pattern. Although the 4+4 pattern (typical in *B.sinica*) was observed in endopod of leg 2 in two specimens of *Bestiolinasarae* sp. n. (left and right leg 2 in paratype NHMW 26309; right leg 2 in paratype 26310), *B.sinica* bears four spinules on the posterior surface of endopod 2 of leg 4, while in *Bestiolinasarae* sp. n. it is always naked.

*Bestiolinasarae* sp. n. is a component of plankton of tropical waters (28.7–28.8 °C). It was found in brackish waters with low salinity (23.0–23.9 pt), dissolved oxygen from 6.4 to 6.7 mg/L, and primary productivity (in terms of chorophyll-*a*) with 2.8–3.0 µg/l. Where *B.sarae* was collected, light penetration of the water was low (Secchi disk depth 3.6–4.8 m).

In the present study, *B.sarae* sp. n. showed a wide range of densities, from 3 to 624 individuals/m^3^. As the species was found in all six localities, separated by up to 500 km from each other, it seems to be widely distributed in the area and could represent a typical copepod of the zooplankton of the Panama Bight. Due to the climatological and oceanographical characteristics of the study area (Dimar 2002; Fernández-Álamo and Färber-Lorda 2006), it seems likely that this species also occurs in coastal waters of the Baudó and Sanquianga ecoregions of the Colombian Pacific (Diaz and Acero 2003) and in other countries of the Eastern Tropical Pacific, such as Panama and Ecuador. It is conceivable that *B.sarae* also occurs in other regions influenced by the El Niño Southern Oscillation phenomenon as result of the tropicalization of species (Carrasco and Santander 1987).

## Conclusions

With the discovery of *Bestiolinasarae* sp. n., the number of species of the genus is increased to nine, with two of them living in coastal waters of the tropical Americas. It is the first representative of the genus in the Eastern Tropical Pacific and seems to be native to the Panama Bight. It seems possible that the species is also distributed in neighbouring coastal waters such as those of Ecuador and Panama, and it might also be expected in other areas influenced by the climatological and oceanographical El Niño Southern Oscillation.

*Bestiolinasarae* sp. n. was probably not detected in the past due to the paucity of surveys in the study area, the use of inappropriately sized zooplankton nets, and the confusion of adults with juvenile stages of other paracalanids. For future studies, we recommend the use of nets with mesh sizes less than 150 µm, which will allow for the collection of small copepods such as *B.sarae* sp. n. and other members of Paracalanidae.

### Key to the identification of females of the genus *Bestiolina*

(Modified from Moon et al. 2010)

**Table:** 

1	Presence of row of spinules on the distal margin of fifth pedigerous somite	**4**
–	Absence of row of spinules on the distal margin of fifth pedigerous somite	**2**
2	Leg 2: presence of spinules on posterior surface of third exopod segment	***Bestiolinasimilis* (Sewell, 1914)**
–	Leg 2: absence of spinules on posterior surface of third exopod segment	**3**
3	Leg 2: presence of spinules on posterodistal surface of second exopod. Body size > 1 mm	***B.inermis* (Sewell, 1912)**
–	Leg 2: absence of spinules on posterodistal surface of second exopod. Body size < 1 mm	***B.arabica* (Ali et al., 2007)**
4	Leg 2: absence of spinules on posterodistal surface of first exopod segment	**5**
–	Leg 2: presence of spinules on posterodistal surface of first exopod segment	**7**
5	Leg 3: presence of spinules on posterodistal surface of second exopod	***B.coreana* (Moon et al., 2016)**
–	Leg 3: absence of spinules on posterodistal surface of second exopod	**6**
6	Leg 4: presence of spinules on posterodistal surface of second endopod segment. Body size almost 1 mm	***B.sinica* (Shen & Lee, 1966)**
–	Leg 4: absence of spinules on posterodistal surface of second endopod segment. Body size less than 0.8 mm	***B.sarae* sp. n.**
7	Leg 4: presence of spinules on posterodistal surface of exopod. Body size > 0.9 mm	***B.amoyensis* (Li & Huang, 1984)**
–	Leg 4: absence of spinules on posterodistal surface of exopod. Body size < 0.7 mm	**8**
8	Leg 3: presence of spinules on posterodistal surface of second and third exopod. Cephalosome fused with first pedigerous somite	***B.zeylonica* (Andronov, 1972)**
–	Leg 3: absence of spinules on posterodistal surface of second and third exopod. Cephalosome separated from first pedigerous somite	***B.mexicana* (Suárez-Morales & Almeida-Artigas, 2016)**


## Supplementary Material

XML Treatment for Bestiolina sarae
